# NO_x_-, IL-1β-, TNF-α-, and IL-6-Inhibiting Effects and Trypanocidal Activity of Banana (*Musa acuminata*) Bracts and Flowers: UPLC-HRESI-MS Detection of Phenylpropanoid Sucrose Esters

**DOI:** 10.3390/molecules24244564

**Published:** 2019-12-13

**Authors:** Louis P. Sandjo, Marcus V. P. dos Santos Nascimento, Milene de H. Moraes, Luiza Manaut Rodrigues, Eduardo M. Dalmarco, Maique W. Biavatti, Mario Steindel

**Affiliations:** 1 Department of Chemistry, Federal University of Santa Catarina, 88040-900 Florianópolis, Brazil; 2 Department of Clinical Analysis, Centre of Health Sciences, Federal University of Santa Catarina, 88 040 970 Florianópolis, Brazil; mmmarcusster@gmail.com (M.V.P.d.S.N.); eduardo.dalmarco@ufsc.br (E.M.D.); 3 Laboratory of Protozoology, Department of Microbiology, Immunology and Parasitology Centre of biological sciences, Federal University of Santa Catarina, 88040-900 Florianópolis, Brazil; milenehoehr@gmail.com (M.d.H.M.);; 4 Department of Pharmaceutical Sciences, Centre of Health Sciences, Federal University of Santa Catarina, 88040-900 Florianópolis, Brazil; maique.biavatti@ufsc.br

**Keywords:** banana inflorescences, anti-inflammatory activity, antiparasitic activity, UPLC-ESI-MS, arylpropanoid sucroses

## Abstract

Banana inflorescences are a byproduct of banana cultivation consumed in various regions of Brazil as a non-conventional food. This byproduct represents an alternative food supply that can contribute to the resolution of nutritional problems and hunger. This product is also used in Asia as a traditional remedy for the treatment of various illnesses such as bronchitis and dysentery. However, there is a lack of chemical and pharmacological data to support its consumption as a functional food. Therefore, this work aimed to study the anti-inflammatory action of *Musa acuminata* blossom by quantifying the cytokine levels (NO_x_, IL-1β, TNF-α, and IL-6) in peritoneal neutrophils, and to study its antiparasitic activities using the intracellular forms of *T. cruzi*, *L. amazonensis*, and *L. infantum*. This work also aimed to establish the chemical profile of the inflorescence using UPLC-ESI-MS analysis. Flowers and the crude bract extracts were partitioned in dichloromethane and *n*-butanol to afford four fractions (FDCM, FNBU, BDCM, and BNBU). FDCM showed moderate trypanocidal activity and promising anti-inflammatory properties by inhibiting IL-1β, TNF-α, and IL-6. BDCM significantly inhibited the secretion of TNF-α, while BNBU was active against IL-6 and NO_x_. LCMS data of these fractions revealed an unprecedented presence of arylpropanoid sucroses alongside flavonoids, triterpenes, benzofurans, stilbenes, and iridoids. The obtained results revealed that banana inflorescences could be used as an anti-inflammatory food ingredient to control inflammatory diseases.

## 1. Introduction

Several nutritionists and food chemists have been working intensively with non-conventional food plants (NCFPs). Fruits, seeds, roots, flowers, leaves, or entire plants can constitute these foods and, in most cases, they represent a new cultural food value for various communities [1]. However, some NCFPs are only occasionally consumed, while others are actually daily dishes in rural localities. The intensification of the use of nonconventional food plants has been widely influenced by socioeconomic conditions (decrease of buying power), taste, and natural calamities such as unfavorable dry seasons and flood [2]. Therefore, these plants play a crucial role as an alternative food supply able to prevent nutritional problems and hunger [2]. The race to introduce NCFPs as food alternatives has sometimes meant that scientific investigations to support the safety of consumers have been lacking. Plants newly considered NCFPs must be chemically studied in order to rule out the presence of toxins, mutagenic, carcinogenic, and teratogenic substances, and other harmful metabolites. Chemical study can also bring to light substances with beneficial biological activity and good nutritional value. This last indication was the main focus of this work; the present study aimed to establish the chemical profile of *Musa acuminata* inflorescences, which are widely consumed in rural areas of Brazilian Amazonia, coast regions of Parana, and São Paulo [3]. “Salpição de mangará” and “amilácea”, meaning, respectively, “salad of banana heart” and “starchy”, are the two principal dishes made of *Musa* flower [1]. Apart from being consumed, *Musa* inflorescences and other parts of the plant are used popularly in Asia and Africa to cure dysentery, hypertension, diabetes, ulcers, and bronchitis [4]. In South Africa, a decoction of this flower is taken three times a day to normalize blood pressure [5]. Various biological investigations have been previously performed with banana flowers of different *Musa* species. Among these, the most studied are the inflorescences from *Musa paradicicus*, *Musa* sp var Nanjangud rasa bale, and *Musa acuminata*, which, respectively, have shown antioxidant, antihyperglycaemic, and anticholesterolaemic activities [6,7,8]. To date, chemical investigations conducted on *Musa acuminata* have focused intensively on rhizomes, unripe fruits, and tissues, from which phenalenones and phenylphenalenones have been identified as the predominant components [9,10,11]. These compounds have been described to have a phytoalexin role for *Musa* species against pathogenic crop microorganisms [9,10,11].

As the literature search on *Musa acuminata* inflorescences revealed few pharmacological and phytochemical studies, this work aimed to biologically and chemically investigate the abovementioned plant material. A part of our research goal was to search for and select non-conventional edible plants from Brazilian Amazonia that are potential anti-inflammatory and antiparasitic treatments. Diseases caused by parasites or leading to inflammation are a health problem in this area, which is why this work aimed to search in the crude extract of the banana inflorescence for natural products possessing an inhibitory effect against inflammatory mediators, including IL-1B, NO_x_, IL-6, and TNF-α. More importantly, we evaluated their antiparasitic activities against the intracellular forms of *Leishmania amazonensis*, *L. infantum*, and *Trypanosoma cruzi* amastigotes grown in human monocytic THP-1 cells. The results obtained prompted us to establish the chemical profile of each fraction based on their UPLC-ESI-MS/MS dereplication.

## 2. Results

Flowers and bracts of *M. acuminata* blossom were separated and extracted with methanol. Because of the presence of insoluble precipitate whether in water or in polar organic solvents, both crude extracts were separately poured into water and were extracted successively with dichloromethane and *n*-butanol, affording four fractions (flower: FDCM and FNBU, respectively; bract: BDCM and BNBU, respectively).

### 2.1. Biological Activities

#### 2.1.1. Anti-Inflammatory Activity

##### Cell Viability

**Figure 1:**
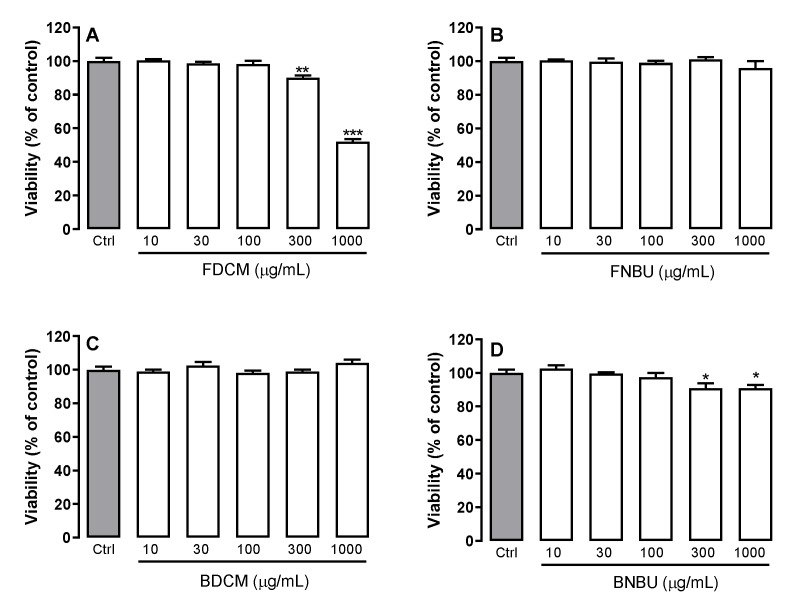
Effects of flower fraction from dichloromethane partition (FDCM) (**A**), flower fraction from *n*-butanol partition (FNBU) (**B**), bract fraction from dichloromethane partition (BDCM) (**C**), and bract fraction from *n*-butanol partition (BNBU) (**D**) on neutrophil viability. Control: peritoneal neutrophils isolated from mice treated only with vehicle; 10–1000: peritoneal neutrophils isolated from mice treated with concentrations of each specific extract ranging from 10 to 1000 µg/mL. Each group represents the mean ± standard error of the mean; *n* = 3/group. * *p* < 0.05, ** *p* < 0.01, and *** *p* < 0.001 compared to the control group (ctrl).

The effects of the organic fractions on isolated mouse neutrophils were determined by trypan blue exclusion test of cell viability, as shown in Figure 1. FDCM and BNBU showed significant toxicity when tested from 10 to 1000 µg/mL (*p* < 0.05). The other two fractions, FNBU and BDCM, showed no effect on neutrophil viability at the tested concentrations (*p* > 0.05).

##### Inhibition Study on Cytokine Secretion (IL-1β, TNF-α, and IL-6)

**Figure 2:**
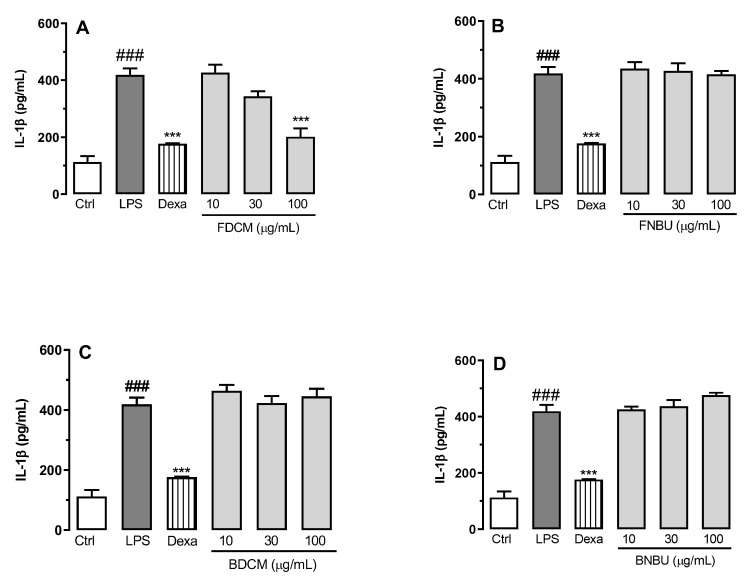
Effect of FDCM (**A**), FNBU (**B**), BDCM (**C**), and BNBU (**D**) on IL-1β secretion by LPS-stimulated peritoneal murine neutrophils. Control: peritoneal neutrophils isolated from mice treated only with vehicle; LPS: peritoneal neutrophils isolated from mice stimulated with LPS and treated with vehicle; 10–100: peritoneal neutrophils isolated from mice stimulated with LPS and treated with concentrations of each specific extract ranging from 10 to 100 µg/mL. Each group represents the mean ± standard error of the mean; *n* = 3/group. ### *p* < 0.001 compared to the Ctrl group. *** *p* < 0.001 compared to the Ctrl group.

On the basis of the results obtained from the cell viability assay, inhibition effects of the fractions were evaluated on pro-inflammatory mediators at non-cytotoxic concentrations. Only FDCM showed significant inhibition of IL-1β levels, reducing the levels of this inflammatory mediator by 51.9% ± 7.2% (*p* < 0.001) of when tested at 100 µg/mL (Figure 2A). Other fractions in turn weakly affected secretion of this inflammatory mediator (*p* > 0.05) (Figure 2B–D).

**Figure 3:**
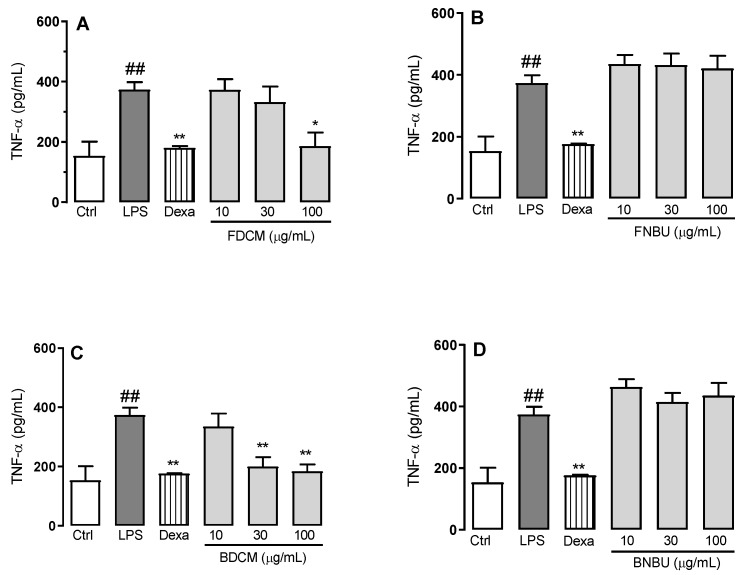
Effect of FDCM (**A**), FNBU (**B**), BDCM (**C**), and BNBU (**D**) on TNF-α secretion by LPS-stimulated peritoneal murine neutrophils. Control: peritoneal neutrophils isolated from mice treated only with vehicle; LPS: peritoneal neutrophils isolated from mice stimulated with LPS and treated with vehicle; 10–100: peritoneal neutrophils isolated from mice stimulated with LPS and treated with concentrations of each specific extract ranging from 10 to 100 µg/mL. Each group represents the mean ± standard error of the mean; *n* = 3/group. ## *p* < 0.001 compared to the Ctrl group. * *p* < 0.01 and ** *p* < 0.001 compared to the Ctrl group.

While the level of TNF-α was decreased by 46.1% ± 8.0% (*p* < 0.05) when neutrophils were treated with FDCM at 100 µg/mL (Figure 3A), BDCM at concentrations of 30 and 100 µg/mL inhibited the secretion of the same cytokine by 46.5% ± 8.6% and 50.7% ± 6.2%, respectively (*p* < 0.01) (Figure 3C). On the other hand, FNBU and BNBU did not reduce this inflammatory mediator (*p* > 0.05) (Figure 3B,D).

**Figure 4:**
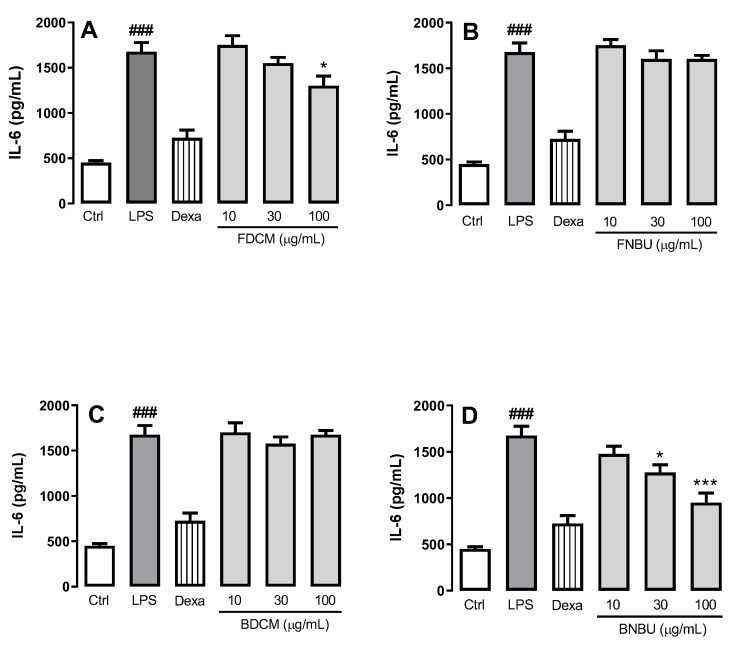
Effect of FDCM (**A**), FNBU (**B**), BDCM (**C**), and BNBU (**D**) on IL-6 secretion by LPS-stimulated peritoneal murine neutrophils. Control: peritoneal neutrophils isolated from mice treated only with vehicle; LPS: peritoneal neutrophils isolated from mice stimulated with LPS and treated with vehicle; 10–100: peritoneal neutrophils isolated from mice stimulated with LPS and treated with concentrations of each specific extract ranging from 10 to 100 µg/mL. Each group represents the mean ± standard error of the mean; *n* = 3/group. ### *p* < 0.001 compared to the Ctrl group. * *p* < 0.01 and *** *p* < 0.001 compared to the Ctrl group.

Weak reductions of the IL-6 level were observed with FDCM (22.3% ± 6.4%) (*p* < 0.05) at 100 µg/mL (Figure 4A), and with BNBU at 30 and 100 µg/mL (23.8% ± 5.0% and 43.2% ± 6.2%, respectively) (*p* < 0.05) (Figure 4A,D). FNBU and BDCM showed no inhibition effect against this inflammatory cytokine (*p* > 0.05) (Figure 4B,C).

##### Measurement of NO_x_ Production by Mouse Neutrophils

**Figure 5:**
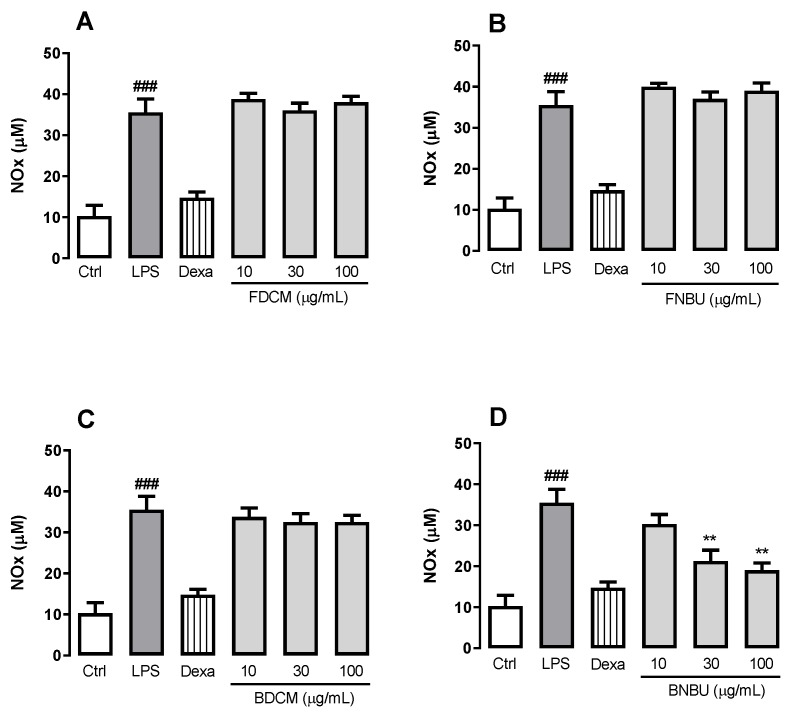
Effect of FDCM (**A**), FNBU (**B**), BDCM (**C**), and BNBU (**D**) on NO_x_ secretion by LPS-stimulated peritoneal murine neutrophils. Control: peritoneal neutrophils isolated from mice treated only with vehicle; LPS: peritoneal neutrophils isolated from mice stimulated with LPS and treated with vehicle; 10–100: peritoneal neutrophils isolated from mice stimulated with LPS and treated with concentrations of each specific extract ranging from 10 to 100 µg/mL. Each group represents the mean ± standard error of the mean; *n* = 3/group. ### *p* < 0.001 compared to the Ctrl group. ** *p* < 0.01 compared to the Ctrl group.

FDCM, FNBU, and BDCM were unable to reduce the levels of NO_x_ secretion regardless of the concentrations tested (*p* > 0.05) (Figure 5A–C), whereas BNBU was able to reduce the production of this inflammatory mediator by 40.2% ± 7.6% and 46.5% ± 5.2%, respectively, (*p* < 0.01) when tested at 30 and 100 µg/mL (Figure 5D).

The relationship between the inflammatory cytokines produced by parasites during infection and their virulence [12,13,14,15] prompted us to evaluate all the fractions against the intracellular forms of *T. cruzi*, *L. amazonensis*, and *L. infantum* amastigotes, as well as the viability of the THP-1 cells (human monocytic cell line) used as host.

#### 2.1.2. Antitrypanosomal and Antileishmanial Activities

FDCM also revealed low toxicity to THP-1 cells with a CC_50_ value of 341.5 µg/mL, leading to a selectivity index of 9.14 (Table 1). As the cytotoxicity of FDCM was nearly 300 µg/mL, a non-toxic concentration of 50 µg/mL was considered to preserve the viability of the macrophage, with the aim of observing significant antiparasitic activity.

FDCM inhibited 90.37% of *T. cruzi* growth, corresponding to an IC_50_ value of 37.35 µg/mL. It also showed weak effects against *L. amazonensis*, and *L. infantum* amastigotes, with inhibition of 37.13% and 11.04%, respectively. The remaining fractions showed weak to no activity against *T. cruzi* and the studied *Leishmania* species.

### 2.2. Chemistry

**Figure 6:**
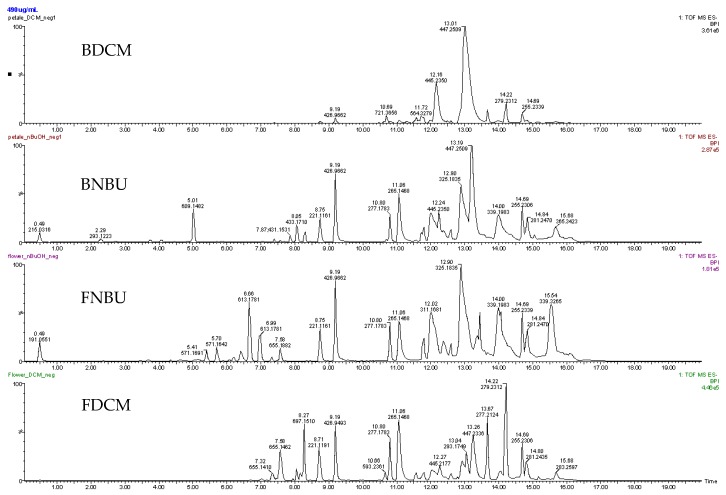
Spectra of the dichloromethane and *n*-butanol flower (FDCM and FNBU, respectively) and bract (BDCM and BNBU, respectively) fractions.

The hyphenated techniques UPLC-ESIMS and UPLC-ESI-MS^2^ were used to establish the chemical compositions of FDCM, FNBU, BDCM, and BNBU, as they showed promising biological activity against inflammation mediators. Their constituents were exclusively sensitive to the negative ionization mode, and the base peak ionization (BPI) was used as the acquisition parameter of the chromatograms (Figure 6). An error equal to or less than ±5 ppm was considered for the determination of the molecular formula. FDCM showed in its LC-ESI-MS chromatogram the presence of 15 metabolites, while 23 components were detected in FNBU (Tables S1 and S2).

The first two metabolites of FDCM were detected at 6.66 and 6.99 min with the same mass value, *m/z* 613.1731, corresponding to the elemental composition C_27_H_34_O_16_. Due to their low quantity in the extract, no fragment was obtained in the tandem mass analysis. However, on their MS spectra, a fragmentation pattern corresponding to a loss of ketene (42 Da) was observed. The obtained *m/z* 571.1544 fragment is a typical in-source-generated product, of which the mechanism has previously been studied and reported [16]. A literature search of this elemental composition led to the structure of four isomeric acetylated sucroses, namely mumeose G, mumeose S, mumeose H, and tomenside A. Up to now, these arylpropanoid sucroses have never been previously reported in banana species.

Two other metabolites appeared in the chromatogram at tR 7.32 and 7.58 min with the same mass value, *m/z* 655.1874, corresponding to C_29_H_36_O_17_. Both were different from the precedents at 6.66 and 6.99 min by 42 Da, indicating their acetylated derivatives. The tandem mass data of *m/z* 655.1874 showed ions consistent with structures of coumaric acid (*m/z* 163.0352), monoglycosylated coumaric acid (*m/z* 349.0909), and triacetylated disaccharide (*m/z* 467.1384) (Figure S1). The metabolites at tR 7.32 and 7.58 min gave an almost similar fragmentation pattern, permitting their isomeric relationship to be deduced. A literature search of C_29_H_36_O_17_ led to the structures mumeose I, mumeose L, mumeose Q, mumeose U, and mumeose T, previously obtained from the flower buds of *Prunus mume* [17,18]. The exact structures of the compounds could not be determined based on the mass spectrometric data. However, the *m/z* 349.0909 fragment (Figure S1), corresponding to the monoacetylated glycosyl coumaric acid ion, suggested muneose L and U as potential structures of the metabolite detected at 7.32 min. The isomer (tR 7.58 min) revealed in its MS^2^ spectrum (Figure S1) a fragment at *m/z* 391.0948, leading to the structure of 4,6,2’,6’-*O*-tetraacetyl-3-*O*-p-coumaroylsucrose. This compound was previously obtained from the fruits of *Prunus jamasakura* [19].

Four more compounds were detected with the same mass value, *m/z* 697.1997, at 7.84, 8.05, 8.16, and 8.27 min. The molecular formula was C_31_H_38_O_18_, different from that of muneose L and U by 42 Da, corresponding to an acetyl group. This observation indicated *m/z* 697.1997 to be an acetylated derivative of 4,6,2’,6’-*O*-tetraacetyl-3-*O*-*p*-coumaroylsucrose. These four metabolites showed almost similar features in their tandem mass spectra. However, the one at 7.94 min gave a fragment ion at *m/z* 391.0988 instead of *m/z* 349.0870, suggesting the presence of only an acetyl group on the pentose unit. The aforementioned information led to the structure of two positional isomers, mumeose V and mumeose D. Both compounds were previously identified from the flower buds of *Prunus mume* [17]. The remaining metabolites gave in their MS/MS spectra an *m/z* 391.0988 fragment ion alongside *m/z* 349.0870, both supporting the presence of two acetyl groups on the pentose, as found in 1,6,2’,6’-*O*-tetraacetyl-3-*O*-trans-*p*-coumaroylsucrose. This elemental composition together with the obtained fragment ions led to the structures of four positional isomers, namely prunose I or mumeose N, or mumeose M or mumeose O. These metabolites were all previously obtained from the flower buds of *Prunus mume* [18].

Metabolites related to fatty acids were characterized from 14.22 to 15.68 min based only on their chemical compositions, because no fragments were observed in their tandem mass spectra.

LCMS analysis of FNBU showed the presence of 22 metabolites, among which quinic acid was detected at 0.49 min with an *m/z* 191.0551 [C_7_H_12_O_6_-H]^−^. Characterization of some components was made possible by the interpretation of their tandem mass data compared to those reported in the literature. Thus, the peak at 2.18 min showed a mass value of *m/z* 487.1465, corresponding to [C_21_H_28_O_13_-H]^−^. Its tandem mass data displayed ions at *m/z* 307.0706, 163.0431, and 145.0352, corresponding respectively to [M-H-180]^−^, [M-H-2x162]^−^, and [M-H-180-162]^−^. The diminution of the precursor mass value by 180 Da and 162 Da occurred when the *m/z* 487.1465 fragment lost a hexopyranose unit or a hexofuranose unit. The *m/z* 163.0431 and 145.0352 fragments corresponding to the coumaric acid ion were formed after the loss of sucrose [20]. A literature search led to the structure of three isomeric metabolites, among which 3-*O*-*p*-coumaroylsucrose was assigned as the structure. This metabolite was previously identified in dried fruits of *Prunus domestica* [21].

Another metabolite was found at 2.37 min with *m/z* 487.1465 [C_21_H_28_O_13_-H]^−^, suggesting an isomer of 3-*O*-*p*-coumaroylsucrose. Tandem mass of the *m/z* 487.1465 data showed fragment ions at *m/z* 341.0868 and 179.0580, which were formed after the loss of a deoxyhexose (146 Da) and a disaccharide (deoxyhexose + hexose, 308 Da), respectively. The *m/z* 179.0580 aglycone was characterized as caffeic acid, and the structure of this metabolite was assigned as cistanoside F. Its fragmentation behavior was similar to that previously reported [22].

The metabolite at 3.43 min with *m/z* 529.1561 [C_23_H_30_O_14_-H]^−^ gave in its MS/MS spectrum ions at *m/z* 487.1449, 469.1057, and 341.0905, formed after the precursor lost an acetyl group (42 Da), acetic acid (60 Da), and acetyldeoxyhexose (188 Da), respectively. The *m/z* 341.0905 fragment ion suggested this metabolite to be related to cistanoside F. Based on the aforementioned information, the structure of *m/z* 529.1561 was assigned to be a derivative of acetyl cistanoside F.

Two other isomers were observed at 3.69 and 4.35 min with the mass value *m/z* 529.1561 [C_23_H_30_O_14_-H]^−^. These compounds differed from 3-*O*-*p*-coumaroylsucrose by 42 Da, suggesting an acetyl derivative. Both isomers also showed a fragmentation pattern similar to 3-*O-p*-coumaroylsucrose with fragment ions at *m/z* 487.1330 [M-H-ketene (42 Da)]^−^, 307.0742 [M-H-acetylhexose (222 Da)]^−^, 163.0405 [M-H-sucrose]^−^, and 145.0352 [M-H-sucrose-H_2_O]^−^. The abovementioned data led to the structure being related to mumeose A and acetyl 3-*O*-*p*-coumaroylsucrose.

Four peaks at 4.60, 5.15, 5.41, and 5.70 min showed the same mass value, *m/z* 571.1642 [C_25_H_32_O_15_-H]^−^. Their molecular formulas differed from that of muneose A by 42 Da, consistent with an acetylated derivative. These compounds gave similar fragment ions at *m/z* 529.1467, 511.1470, 307.0814, 163.0405, and 145.0352 in their tandem mass spectra. The *m/z* 529.1467 and 511.1470 ions were formed from the loss of an acetyl group (42 and 60 Da). The *m/z* 307.0814 ion was produced from the precursor after the loss of a diacetylhexose (264 Da). The structure of the aglycone was also assigned as coumaric acid based on the presence of the *m/z* 163.0405 and 145.0352 ions [20]. On the basis of the abovementioned information, the structures of these metabolites were assigned as positional isomers of mumeose B, P, or R, previously isolated from the flower buds of *Prunus mune* [17].

Four other positional isomers were also found in this fraction at 6.18, 6.40, 6.66, and 6.99 min (*m/z* 613.1731 [C_25_H_32_O_15_-H]^−^). These compounds contained three acetyl groups as their mass value differed from *m/z* 571.1642 by 42 Da, indicating a triacetyl 3-*O*-*p*-coumaroylsucrose derivative. All the *m/z* 613.1731 precursors gave in their MS/MS spectra similar fragments at *m/z* 571.1592, 553.1507, 529.1467, 511.1423, 349.0909, 307.0814, 163.0378, and 145.0278, while the *m/z* 571.1592 and 553.1507 fragments corresponded respectively to [M-ketene (42 Da)-H]^−^ and [M-acetic acid (60 Da)-H]^−^. The *m/z* 529.1467 and 511.1423 fragments were consistent with [M-H-2xketene(84 Da)]^−^ and [M-H-2xacetic acid (120 Da)]^−^. The *m/z* 349.0909 [M-264 (diacetylhexose)-H]^−^ ion suggested the presence of an acetyl group on the sugar directly attached to the aglycone. Furthermore, this fragment lost a ketene (42 Da) to afford an *m/z* of 307.0814. As observed in the MS spectrum of the abovementioned metabolites, the aglycone was coumaric acid, consistent with an *m/z* 163.0378 and its dehydrated *m/z* 145.0278 fragment ion [20]. Because the position of the acetyl groups could not be determined using MS data, these four metabolite structures were deduced to be related to tomenside B based on the aforementioned information. Tomenside B is a triacetylated phenylpropanoid sucrose previously obtained from *Prunus tomentosa* leaves [23].

LCMS data of FNBU also showed two metabolites at 7.32 and 7.58 min with the same mass value, *m*/*z* 655.1882 [C_29_H_36_O_17_-H]^−^. Their structures were found to be related to muneose L and U for the metabolite at 7.32 min, while its isomer at tR 7.58 min was characterized as 4,6,2’,6’-*O*-tetraacetyl-3-*O-p*-coumaroylsucrose. A procyanidin derivative and a glycosylated flavonoid were found at *m/z* 1197.2529 [2xC_32_H_24_O_12_+HCO_2_]^−^ and 637.1405 [C_27_H_28_O_15_+HCO_2_]^−^, respectively. The lack of fragmentation was presumably due to their low quantities in the fraction. A pentacyclic triterpenic acid and a caffeate of betuline were also detected at 13.45 and 14.07 min with the mass values of *m/z* 455.3515 [C_30_H_48_O_3_-H]^−^ and 609.4099 [C_39_H_56_O_5_-H]^−^, respectively.

LCMS analysis of these BDCM fraction showed the presence of nine compounds, among which four were characterized (Table S2). The structure of the first metabolite at 7.36 min with an *m/z* 177.0550 [C_10_H_10_O_3_-H]^−^ was assigned as coumaric acid methyl ester or 4-methoxycinnamic acid. No fragment was found to completely elucidate its structure.

The compound at 10.69 min showed ions at *m/z* 721.3635 [M+HCO_2_]^−^ and *m/z* 675.3600 [M−H]^−^, corresponding to C_33_H_56_O_14_. This compound afforded on its MS/MS spectrum a fragment ion at *m/z* 593.3170, consistent with the loss of 2-methylbuta-1,3-dien-1-one (82 Da). The *m/z* 723.3801 [C_33_H_58_O_14_+HCO_2_]^−^ precursor ion did not provide a fragment ion; however, the structures of 3’-*O*-isobutyryl-3-*O*-isovaleryl-2-*O*-lauroylsucrose and 2,3,4-tri(5-methylhexanoyl)-α-d-glucopyranosyl-β-d-fructofuranoside were suggested. The metabolites between 11.76 and 13.78 min did not furnish any fragment ions, but the literature indicated a structural relationship with stilbene. This group of metabolites has already been reported in Musaceae [24].

BNBU’s LCMS data (Table S2) showed a peak at 2.29 min with *m/z* 293.1223, corresponding to [C_12_H_22_O_8_-H]^−^. A literature search provided γ-methyl-δ-hydroxy-pentanoic acid β-d-glucopyranoside as a reliable structure [25].

The *m/z* 609.1482 peak at 5.01 min [C_27_H_30_O_16_-H]^−^ gave fragment ions at *m/z* 581.1510 and 461.1089. The *m/z* 581.1510 fragment was formed when the precursor lost CO (28 Da); this fragment in turn dehydrated and afforded *m/z* 461.1089 after a retro-Diels–Alder rearrangement (Figure S2). The structure of this metabolite was deduced to be 6,8-di-*C-*β*-*d-glucopyranosyl-luteoline, previously detected in *Citrus* peels [26].

The peak at 7.87 min with *m/z* 431.1531 [C_19_H_28_O_11_-H]^−^ showed on its tandem mass spectrum ions at *m/z* 349.0985 [M-CH_3_CH_2_OH-2H_2_O-H]^−^, 331.0892 [M-CH_3_CH_2_OH-3H_2_O-H]^−^, and 113.0320 [M-aglycone-H_2_O-H_2_CO-H]^−^ (Figure S3). This information led to the structure of diffusosides A or B, two diastereomeric iridoids previously obtained from *Hedyotis diffusa* [27].

The peak at 8.05 min with an *m/z* of 433.1710 [C_19_H_30_O_11_-H]^−^ showed in its MS/MS spectrum a fragmentation pattern similar to that of the precedent metabolite, indicating another iridoid derivative. The *m/z* 351.1140 ion was obtained after the precursor eliminated CH_3_CH_2_OH (46 Da) and 2H_2_O (36 Da). The *m/z* 333.1045 ion was formed after the removal of CH_3_CH_2_OH (46 Da) and three molecules of H_2_O (54 Da), while the *m/z* 113.0298 ion was produced from the loss of the aglycone (272 Da), H_2_O (18 Da), and CH_2_CO (30 Da).

This information, together with that presented in Figure S4, indicated the structure of 7-*O*-ethylmorroniside. Another iridoid was found at 8.31 min with an *m/z* of 435.1851 [C_19_H_32_O_11_-H]^−^. This metabolite was heavier than 7-*O*-ethylmorroniside by a double bond equivalence. No structure matched the tandem mass data; however, the similarity between its fragment ions and those of 7-*O*-ethylmorroniside enabled the deduction that this metabolite was an iridoid derivative.

Two other metabolites were found at 8.75 and 13.23 min with *m/z* values of 221.1178 and 447.2509, respectively. The lack of fragmentation limited their structural assignment; however, a literature search indicated that these compound structures were related to those of an alkylated phenol and a stilbene, respectively.

### 2.3. Discussion

The cytotoxic effect of the studied extracts on the isolated mouse neutrophils showed that FDCM and BNBU reduced these cells’ viability at concentrations equal to or greater than 300 μM. Alongside acetylated arylpropanoid sucroses, FDCM was also composed of fatty acids and other phenolics, while FNBU was formed of flavonoids, triterpenes, cyclohexanetetrol, and a low quantity of acetylated arylpropanoid sucroses (Table 2). The most concentrated sucrose, at 8.27 min (*m/z* 697.1997), and its positional isomers were presumably responsible for the cytotoxicity observed against neutrophil cells. However, their weak concentration in FNBU might support why this fraction lacked cytotoxic activity. Interestingly, compounds related to 3-phenylpropanoid-triacetyl sucrose esters, such as tomensides A–D and numeose C, demonstrated cytotoxicity against four human cancer cell lines in a previous study, although no information was provided about their selectivity towards normal cells [23].

The LCMS data of BDCM showed the presence of arylpropanoids, glycolipids, arylbenzofurans, fatty acids, and stilbenes; among them, an *m/z* 447.2509 stilbene derivative was the major component. No cytotoxicity was observed for this fraction. However, neutrophil cells responded slightly to BNBU, which was rich in glycolipids, stilbenes, flavonoids, arylbenzofurans, other phenolics, and iridoids, among which 6,8-di-*C*-glycosylated luteolin and an *O*-acyl glycoside were the main components. A previous study revealed that 6,8-di-*C*-β-d-glucopyranosyl-luteolin (lucenin-2) is weakly or not cytotoxic against five cancer cell lines [28]. Therefore, iridoids might be responsible for the cytotoxic effect on neutrophils, since some of these metabolites have been described as antiproliferative agents [29]. No toxicity study was found in the literature on banana inflorescences; however, previous bioassays have shown that its incorporation in rat diets might modulate serum cholesterol and glucose [8].

The anti-inflammatory effect of these fractions was evaluated at concentrations ranging from 10 to 100 µM. A different inhibitory profile was observed when these fractions were tested on the anti-inflammatory mediators IL-1β, TNF-α, NO_x_, and IL-6. FDCM, rich in phenylpropanoid sucroses (*m/z* 613.1731, 655.1882, and 697.1997), fatty acids, and other phenolic compounds, inhibited the mediators IL-1β, TNF-α, and IL-6; its anti-inflammatory activity was presumably related to the presence of these phenolic glycosides. This conclusion is supported by former studies reporting similar metabolites with the same pharmacological property [30]. These arylpropanoid sucroses have also been described as inhibitors of aldose reductase, which is involved in various inflammatory disorders [17,18]. In fact, inhibition of aldose reductase might reduce reactive oxygen species and, therefore, prevent the inflammatory signals induced by cytokines and other factors [17,18]. Despite the presence of these metabolites in FNBU, no inhibition effect was observed on IL-1β, TNF-α, IL-6, and NO_x_ levels. FNBU showed traces of metabolites at *m/z* 613.1731, 655.1882, and 697.1997 in its LCMS data, alongside other arylpropanoid sucroses (3-*O-p*-coumaroylsucrose, cistanoside F, and acetyl cistanoside F derivative), flavonoid derivatives, and pentacyclic triterpenes. These classes of metabolites are recognized to possess anti-inflammatory properties [31,32]. Therefore, the lack of anti-inflammatory activity of FNBU might have been due to the low concentrations of these components, which were not sufficient to produce the expected effect.

BDCM contained a stilbene derivative which, among other metabolites, inhibited only TNF-α. This fraction displayed a chemical profile different from those of FDCM and FNBU, and the lack of sucroses might be why this fraction showed a different inhibition profile. On the other hand, its effect on TNF-α level could have been associated with the presence of a stilbene, of which the analogues, such as resveratrol, are known to be inhibitors of TNF-α [33]. In addition, the presence in BDCM of coumaric acid methyl ester (7.36 min, *m/z* 177.0550) related to the aglycone of the arylpropanoid sucroses could also have contributed to the inhibition of TNF-α levels. The similarity of BNBU and FDCM relied on an unidentified phenolic, although BNBU was able to inhibit the increase of NO_x_ and IL-6 levels. Considering the chemical profiles and the inhibition effects of FNBU and BDCM, iridoids and the phenolic derivative might have been responsible for the anti-inflammatory activity of BNBU. Iridoids structurally related to those found in BNBU, such as morroniside and geniposide, have been described as anti-inflammatory agents, and morroniside in particular is a NO_x_ inhibitor [34]. The presence of a diffusoside derivative and 7-*O*-ethylmorroniside might support the observed anti-inflammatory activity of this fraction. Lucenin-2, a 6,8-di-*C*-glycosylated flavone, could also have contributed to this activity based on previous results describing its anti-inflammatory properties [35].

Since inflammatory cytokines are produced during parasite infections and these cytokines are also manifestly related to their virulence [12,13,14,15], this study also aimed to investigate whether fractions from banana blossom could exert antiparasitic effects against intracellular *T. cruzi*, *L. amazonensis*, and *L. infantum* amastigotes.

As human monocyte THP-1 cells were used as the macrophage, their viability when treated with the only active fraction (FDCM) was evaluated. This fraction was weakly cytotoxic to the THP-1 cell line. As observed with the neutrophil cells, FDCM, composed essentially of phenylpropanoid sucroses, required a high concentration to affect cell viability.

Only FDCM showed antitrypanosomal activity against the intracellular form of *T. cruzi* with a good selectivity index. It has been reported in the literature that human macrophages infected with *T. cruzi* display an increased level of MMP-9, which has a strong relationship with the production of inflammatory cytokines such as IL-1β, TNF-α, and IL-6 [14]. Therefore, the trypanocidal activity observed for FDCM might have had a relationship with its anti-inflammatory effect by inhibiting these three cytokines. In contrast to FDCM, which concomitantly inhibited three cytokines, BDCM solely inhibited the cytokine TNF-α and showed no effect against *T. cruzi*. This observation led to the conclusion that FDCM displayed antitrypanosomal activity, because it could reduce the levels of these three cytokines without inhibiting the level of NO_x_. It has been reported that nitrogen-derived species (NO_x_) have a crucial role for the immune system by protecting cells against intracellular *T. cruzi* infection [36]. Therefore, the selective effect on the cytokines but not NO_x_ is important for antitrypanosomal activity. Nitrogen oxide species chemically specifically modify cysteine-containing proteins in *T. cruzi*, and can potentially interact with the metalloproteins that mediate crucial metabolic processes [36]. This might support why BNBU did not show any trypanocidal activity. None of the fractions were active against the studied *Leishmania* species, indicating that the inhibition of IL-1β, TNF-α, and IL-6 cytokines and NO_x_ species might affect the growth of *Leishmania.*

As this non-conventional food showed various biological benefits, it can be classified as a functional food, although more studies including toxicology and balanced diet studies need to be performed.

## 3. Materials and Methods

### 3.1. Plant Identification

The inflorescences of *Musa acuminata* were collected in Itacorubi/Florianópolis in March 2017. A voucher was deposited under the number RB 02574A in the Jardim Botanico (Botanical Garden) of Rio de Janeiro Herbarium (RB).

### 3.2. Anti-Inflammatory Assays

#### 3.2.1. Mouse Neutrophil Isolation and Primary Culture

Mouse neutrophils were collected from mouse peritoneal leakage and maintained in Dulbecco’s Modified Eagle Medium (DMEM) (Gibco, Grand Island, NY, USA) with 10% fetal bovine serum, 100 U/mL of penicillin, and 100 mg/mL of streptomycin incubated at 37 °C in a humidified CO_2_ incubator. The peritoneal neutrophils were obtained after injection of oyster glycogen into the peritoneal mouse cavity, as described by Silva and co-workers with some modifications [37]. A total 3 mL of oyster glycogen at 1% (*w*/*v*) dissolved in sterile phosphate-buffered saline (PBS) was injected into the peritoneal mouse cavity, and after 4 h the animals were euthanized by overdose of xylazine and ketamine administered intravenously (i.v.). After euthanasia, 3 mL of sterile PBS was injected into the peritoneal cavity and the cavity was massaged for 10 s to suspend the neutrophils. An incision was made using sterile surgical material and the peritoneal leakage was collected in 50 mL sterile tubes and stored immediately in an ice bath. Furthermore, a pool of peritoneally collected neutrophils was made in order to obtain 1 × 10^6^ neutrophils/well. A reduced number of animals were used with respect to the 3Rs (Replacement, Reduction and Refinement) principle [38]. The procedures were approved by the Committee for Ethics in Animal Research from UFSC (Protocol 8665141117) and were in accordance with the National Institutes of Health (NIH) Guide for the Care and Use of Laboratory Animals.

#### 3.2.2. Lipopolysaccharide Stimulation of Isolated Neutrophils

The neutrophils were preincubated after plate distribution with or without different concentrations (10, 30, and 100 µg/mL) of the studied fractions for 1 h, and then the medium was exchanged with fresh DMEM mixed with lipopolysaccharide (LPS) at a final concentration of 5 µg/mL and incubated for 16 h at 37 °C in a CO_2_ incubator (5%).

#### 3.2.3. Cell Viability Assay Using the Isolated Neutrophilis

The extracts were added to each well at different final concentrations (10, 30, 100, 300, and 1000 µM) and incubated for 16 h at 37 °C in a CO_2_ incubator (5%). This procedure was performed after the neutrophils were plated in a 96 well plate with DMEM culture medium enriched with 10% fetal bovine serum and 1% (*w*/*w*) penicillin/streptomycin. The entire experiment was conducted in triplicate and repeated on two different days of experimentation. The viability of the neutrophils after treating with the blossom fractions was evaluated using the colorimetric (3-(4,5-dimethylthiazol-2-yl)-2,5-diphenyltetrazolium bromide) MTT assay. The supernatant was discarded after incubation for 16 h and MTT solution (5 mg/mL) was added to each well, followed by incubation for a further 3 h at 37 °C in a CO_2_ incubator (5%). The medium was then discarded again and dimethylsufoxide (DMSO) was added to dissolve the formazan dye. The optical density was checked at 540 nm using an ELISA reader (Infinite M200, Tecan, Männedorf, Switzerland).

#### 3.2.4. Cell Inflammation Assay on Isolated Neutropils

In order to evaluate the effect of the standards and the fractions on inflamed ex vivo mouse neutrophils, cells were designated to different groups (*n* = 4/group) consisting of the following: (a) blank control (Ctrl, uninflamed neutrophils), cells treated only with vehicle; (b) negative control (LPS, lipopolysaccharide-inflamed neutrophils), cells stimulated only with LPS (5 µg/mL); (c) positive controls (dexamethasone: Dexa, reference anti-inflammatory drug treatment), cells pre-treated with Dexa (10 μM) and after 0.5 h stimulated with LPS (5 μg/mL); and (d) experimental groups (studied extracts), cells pre-treated with the extracts at 10, 30, and 100 µg/mL and stimulated after 0.5 h with LPS (5 μg/mL). All experimental groups were incubated for 16 h at 37 °C in a CO_2_ atmosphere (5%). The supernatant was collected for further inflammatory analysis and comparisons (NO_x_, IL-1β, TNF-α, and IL-6).

#### 3.2.5. Measurement of NO_x_ Production in Neutrophils

The production of NO metabolites by mouse neutrophils (*n* = 10 per experiment) was determined using Griess reagent. Measures of 100 μL of the Griess reagent were mixed with 50 μL of cell supernatant and incubated for 40 min at 37 °C. Absorbance at 540 nm was measured with interpolation from the nitrite standard curve (0–20 μM), and the results are expressed in μM.

#### 3.2.6. Quantification of Pro-Inflammatory Cytokines Levels (IL-1β, TNF-α, and IL-6) in Neutrophils

The interleukin-1β (IL-1β), tumoral necrosis factor alpha (TNF-α), and interleukin 6 (IL-6) levels in the neutrophil supernatants were quantified as follows. The supernatant was removed and submitted to determination of the concentrations of IL-1β, TNF-α, and IL-6 using a commercially available enzyme-linked immunosorbent assay kit (Peprotech, Rocky Hill, NJ, USA) according to the manufacturer’s instructions. Cytokine level was estimated by interpolation from the standard curve and the results are expressed in pg/mL.

### 3.3. Antiparasitic Assays

#### 3.3.1. In Vitro Antitrypanosomal and Antileishmanial Assays

The human macrophage cell line THP-1 (ATCC TIB202) was grown in RPMI-1640 without phenol red (Sigma-Aldrich, St Louis, MO, USA), supplemented with 10% FBS (Life Technologies, Carlsbad, CA), 12.5 mM HEPES, penicillin (100 U/mL), streptomycin (100 μg/mL), and Glutamax (2 mM), at 37 °C in a 5% CO_2_ incubator. Schneider’s insect medium (Sigma Chemical Co., St Louis, MO, USA) supplemented with 5% heat-inactivated FBS and 2% human urine at 26 °C was used to grow *L. amazonensis* MHOM/BR/77/LTB0016 and *L. infantum* (MHOM/BR/74/PP75) promastigotes, expressing β-galactosidase. THP-1 cells (4.0 × 10^4^ per well) were cultivated in 96 well microplates with complete RPMI-1640 medium supplemented with 100 ng/mL of phorbol 12-myristate 13-acetate (PMA) (Sigma Chemical Co.) for 72 h at 37 °C in 5% CO_2_, to allow THP-1 cell differentiation into non-dividing macrophages [39]. Four day old culture promastigotes (4.0 × 10^6^ parasites/mL) were washed twice with phosphate-buffered saline (PBS), pH 7.4, and incubated in RPMI-1640 supplemented with 10% heat-inactivated human B+ serum for 1 h at 34 °C for parasite opsonization. Macrophages were incubated with a parasite/cell ratio of 10:1 for 4 h at 34 °C and 5% CO_2_. Thereafter, non-adherent parasites were removed by washing with PBS solution. Infected cells were incubated with 180 μL of fully supplemented RPMI-1640 medium for another 24 h to allow the transformation of promastigotes into intracellular amastigotes. The β-galactosidase *T. cruzi*, Tulahuén strain was obtained from the Laboratory of Cellular and Molecular Parasitology, Centro de Pesquisas René Rachou, FIOCRUZ, Belo Horizonte. Culture-derived trypomastigotes raised from an infected L929 cell line were used to infect differentiated THP-1 cells (4.0 × 10^4^ cells/well) in 96 well microplates in a parasite/cell ratio of 2:1, and were then incubated overnight at 37 °C in a 5% CO_2_ atmosphere. The medium containing non-internalized parasites was removed and replaced with 180 μL of fresh medium [40]. Samples were solubilized in dimethylsulfoxide (DMSO) Merck^®^ and serially diluted (500 μg/mL to 2 μg/mL). The infected cell monolayer was treated with 50 μg/μL of each sample, in triplicate, followed by incubation for 48 h at 34 °C or 37 °C, 5% CO_2_. After treatment, cells were carefully washed with PBS and incubated for 16 h at 37 °C with 250 μL of chlorophenol red-β-d-galactopyranoside (CPRG) (Sigma-Aldrich Co.) at 100 μM and Nonidet P-40 (NP-40) (Amresco Inc, Solon, OH, USA) 0.1%. Optical density was read at 570/630 nm in an Infinite M200 (Tecan, Grödig, Austria) [41,42]. The concentration of each sample that reduced parasite viability by 50% when compared to untreated control (IC_50_) was estimated by non-linear regression of concentration–response curves. Amphotericin B (Sigma-Aldrich) and benznidazole (Sigma-Aldrich) were used as positive controls for antileishmanial and antitrypanosomal activities, respectively, and DMSO 1% as negative control. The concentrations able to inhibit 50% (IC_50_) of the parasites and the proliferation of THP-1 cells (CC_50_) were used to express the antiparasitic activity and cytotoxicity, respectively. Selectivity index (SI) of each sample was determined by the ratio of CC_50_/IC_50_.

#### 3.3.2. Cell Viability Assay (MTT)

THP-1 cells were grown and cultivated in 96 well microplates (4.0 × 10^4^ cells/well), treated with the compounds serially diluted in concentrations ranging from 2 μg/mL to 500 μg/mL, and incubated for 72 h at 37 °C, 5% CO_2_. The plates were centrifuged (3700×*g*/7 min), the supernatant was removed, and the cells were resuspended in 50 μL of a solution of MTT (Amresco) at 3 mg/mL in saline buffer and incubated for 4 h at 37 °C, 5% CO_2_ before being centrifuged (3700×*g*/7 min), and the formazan salt was solubilized in 100 μL DMSO. Optical density was determined at 540 nm in a Tecan^®^ Infinite M200 spectrophotometer. DMSO 1% (*v*/*v*) and DMSO 50% (*v*/*v*) were the negative and positive controls, respectively. The IC_50_ values were calculated by non-linear regression using the GraphPad Prism program [40].

### 3.4. LCMS analysis

#### 3.4.1. Chemicals

Acetonitrile and formic acid were purchased from Tedia (São Paulo, Brazil). A Milli-Q system (18.2MΩ, Millipore, Simplipak, France) was used to prepare ultrapure water. Syringe filters (13 mm, 0.22 μm) were bought from Analítica (São Paulo, Brazil).

#### 3.4.2. Extraction, Fractionation, and Sample Preparation

The banana inflorescences were separated into bracts (228 g) and flowers (60 g), which were extracted in methanol (500 and 100 mL respectively). Both extractions furnished crude extracts of 70 mg from the petals and 40 mg from flowers. Each crude extract was diluted in water and separated by liquid-liquid non-miscible extraction process with dichloromethane and *n*-butanol. FDCM (5 mg) and FNBU (7 mg) were obtained from the flowers, whereas BDCM (4.8 mg) and BNBU (10 mg) were obtained from the bracts. An total 3 mg of each sample was diluted with 4 mL of acetonitrile and methanol (1:1, *v*/*v*) to afford solutions with concentrations of 750 μg/mL, which were filtered using a 0.22 μm syringe filter.

#### 3.4.3. LC-MS Method

An Acquity UPLC system class H (Waters, Milford, MA, USA) equipped with a photodiode array (PDA) detector, sample manager, and a quaternary solvent manager as well as a BEH C18 column (50 mm, 1.0 mm, particle size 1.7 μm (Waters)) was used for the separation. The column and the sample tray were maintained at temperatures of 40 °C and 20 °C, respectively. A sample volume of 3 μL was subjected to a gradient condition at flow rate of 0.3 mL/min: 95% A (water/formic acid, 99.9/0.1 (*v*/*v*)) and 5% B (acetonitrile); 0–2 min, 95% of A; 2–10 min, 55% of A; 10–15 min, 5% of A; 15–20 min, 95% of A.

A Xevo G2-S QTof (Waters) bearing an electrospray ionization (ESI) probe operating in positive and negative ionization modes was coupled to the UPLC device and used to detect the chemical components of each extract. Nebuliser gas: nitrogen; cone gas flow: 100 L/h; desolvation gas flow: 900 L/h; sampling cone 40 V; source offset 80 V; collision gas: argon; lockspray reference sample was leucine encephalin with reference masses at *m/z* 554.2615 (ESI−). The desolvation and the ionization source were maintained during the analyses at 250 °C and 90 °C, respectively, while the capillary voltage was 3 kV. A range of 25 to 35 eV was used as the collision energy. Data were acquired in a range of 100–1500 Da, at a scan time of 1.0 s over 20 min, and were processed with MassLynx V4.1 (Waters).

Molecular formulas were determined by calculation using MassLynx’s elemental composition tool. The choice of each molecular formula was restricted by a tolerance of 5 ppm between the calculated and the measured mass values.

## 4. Conclusions

This work focused on the anti-inflammatory activities of the fractions from banana flower and bracts against NO_x_ and cytokines, including IL-1β, TNF-α, and IL-6. Their effects on intracellular forms of *T. cruzi*, *L. amazonensis*, and *L. infantum* amastigote were also investigated. Only the flower fraction from the dichloromethane partition showed simultaneous anti-inflammatory and antitrypanosomal activities. None of these fractions displayed antileishmanial activites against *L. amazonensis* and *L. infantum*. Interestingly, the fraction from the dichloromethane partition (FDCM), rich in arylpropanoid sucroses, was the most prominent with respect to the investigated biological activities. Fractions showed different anti-inflammatory activities on the tested cytokines. All fractions showed anti-inflammatory activity against at least one cytokine except FNBU. The chemical profiles established by UPLC-ESI-QTOFMS of FDCM, FNBU, BDCM, and BNBU showed the presence of 15, 22, 8, and 9 metabolites, respectively. LCMS data of FDCM revealed the presence of eight arylpropanoid sucroses alongside one phenolic metabolite, four fatty acids, and two unidentified metabolites. FNBU, on the other hand showed the presence of 16 arylpropanoid sucroses in its LCMS data, together with 1 cyclohexanetetrol acid, 1 phenolic compound, 2 flavonoids, and 2 triterpenes. While the LCMS data of BDCM displayed an arylpropanoid, two glycolipids, two stilbenes, one arylbenzofuran, and two fatty acids, those of BNBU showed the presence of one fatty acid, one glycolipid, one arylbenzofuran, one flavonoid, three iridoids, one stilbene, one phenolic derivative, and one unidentified metabolite. The abovementioned results emphasized the health benefit of this non-conventional food and its chemical composition. Therefore, banana blossom may have applications as a dietary food supplement or as a potential functional ingredient to control inflammation.

## Figures and Tables

**Table 1:** In vitro activity of the inflorescence fractions at concentrations of 50 µg/mL against *Trypanosoma cruzi*, *Leishmania amazonensis*, and *L. infantum* intracellular amastigotes.

	*T. cruzi*		*L. amazonensis*		*L. infantum*		THP-1 (Human Monocyte Cells)	SI
Fractions	% inhibition	IC_50_ (µg/mL)	% inhibition	IC_50_ (µg/mL)	% inhibition	IC_50_ (µg/mL)	CC_50_ (µg/mL)	
FDCM	90.37% ± 1.17%	37.35 ± 0.97	37.13% ± 3.15%	ND	11.04% ± 1.51%	ND	341.50 ± 17.20	9.14
FNBU	NA	ND	1.71% ± 0.62%	ND	NA	ND	ND	ND
BDCM	NA	ND	NI	ND	NA	ND	ND	ND
BNBU	5.37% ± 0.32%	ND	5.52% ± 2.30%	ND	NA	ND	ND	ND
Benznidazole	93.48% ± 1.04%	10.18 ± 0.3	-	-	-	-	>500	>49.11
Amphotericin B	-	-	84.14% ± 1.37%	0.09 ± 0.02	76.42% ± 4.24%	0.11 ± 0.03	-	-

NA: no activity; ND: not determined; SI: selectivity index. IC_50_: the concentration of each sample that reduced parasite viability by 50% when compared to untreated control, estimated by non-linear regression of concentration–response curves.

**Table 2:** The chemical constituents characterized in banana inflorescence fractions.

Group of characterized metabolites	Fractions			
	FDCM	FNBU	BDCM	BNBU
Arylpropanoid sucroses	X	X		
Phenolics	X	X		X
Fatty acids	X		X	
Cyclohexanetetrol		X		
Flavonoids		X		X
Triterpenes		X		
Arylpropanoids			X	
Glycolipids			X	X
Stilbenes			X	X
Arylbenzofurans			X	X
Iridoids				X

## Supplementary Materials

The following are available online, Figure S1: Fragmentation pattern of the metabolites *m*/*z* 655.1874 with retention times 7.32 min (left) and 7.58 min (right), Figure S2: Fragmentation pattern of the metabolite *m*/*z* 609.1482, Figure S3: Fragmentation pattern of *m*/*z* 431.1553, Figure S4: Fragmentation pattern of *m*/*z* 433.1710, Table S1: Chemical constituents of the flower fractions, Table S2: Chemical constituents of the bract fractions.

## References

[1] Nunes H. (2017). PANC gourmet: Ensaios culinários.

[2] Azam F.M.S., Biswas A., Mannan A., Afsana N.A., Jahan R., Rahmatullah M. (2014). Are Famine Food Plants Also Ethnomedicinal Plants? An Ethnomedicinal Appraisal of Famine Food Plants of Two Districts of Bangladesh. Evid Based Complement. Altern. Med..

[3] Kinupp V.F., Lorenzi H. (2014). Plantas Alimentícias Não Convencionais (PANC) no Brasil: Guia de identificação, aspectos nutricionais e receitas ilustradas.

[4] Mathew N.S., Negi P.S. (2017). Traditional uses, phytochemistry and pharmacology of wild banana (*Musaacuminata* Colla). A Review. J. Ethnopharmacol..

[5] Chintamunnee V., Mahomoodally M.F. (2012). Herbal medicine commonly used against non-communicable diseases in the tropical island of Mauritius. J. Herb Med..

[6] China R., Dutta S., Sen S., Chakrabarti R., Bhowmik D., Ghosh S., Dhar P. (2011). In vitro Antioxidant Activity of Different Cultivars of Banana Flower (*Musa paradicicus* L.) Extracts Available in India. J Food Sci..

[7] Ramu R., Shirahatti P.S., Dhanabal S.P., Zameer F., Dhananjaya B.L., Prasad M.N.N. (2017). Investigation of Antihyperglycaemic Activity of Banana (*Musa* sp. Var. Nanjangud rasa bale) Flower in Normal and Diabetic Rats. Pharm. Mag..

[8] Liyanage R., Gunasegaram S., Visvanathan R., Jayathilake C., Weththasinghe P., Jayawardana B.C., Vidanarachchi J.K. (2016). Banana Blossom (*Musa acuminate* Colla) Incorporated Experimental Diets Modulate Serum Cholesterol and Serum Glucose Level in Wistar Rats Fed with Cholesterol. Cholesterol..

[9] Otálvaro F., Nanclares J., Vásquez L.E., Quiñones W., Echeverri F., Arango R., Schneider B. (2007). Phenalenone-type compounds from *Musa acuminata* var. “Yangambi km 5” (AAA) and their activity against *Mycosphaerella fijiensis*. J. Nat. Prod..

[10] Hölscher D., Buerkert A., Schneider B. (2016). Phenylphenalenones Accumulate in Plant Tissues of Two Banana Cultivars in Response to Herbivory by the Banana Weevil and Banana Stem Weevil. Plants.

[11] Kamo T., Kato N., Hirai N., Tsuda M., Fujioka D., Ohigashi H. (1998). Phenylphenalenone-type Phytoalexins from Unripe Buñgulan Banana Fruit. Biosci Biotechnol Biochem..

[12] Oliveira W.N., Ribeiro L.E., Schrieffer A., Machado P., Carvalho E.M., Bacellar O. (2014). The role of inflammatory and anti-inflammatory cytokines inthe pathogenesis of human tegumentary leishmaniasis. Cytokine.

[13] Morgado F.N., de Carvalho L.M.V., Leite-Silva J., Seba A.J., Pimentel M.I.F., Fagundes A., Madeira M.F., Lyra M.R., Oliveira M.M., Schubach A.O. (2018). Unbalanced inflammatory reaction could increase tissue destruction and worsen skin infectious diseases—a comparative study of leishmaniasis and sporotrichosis. Sci. Rep..

[14] Vazquez B.P., Vazquez T.P., Miguel C.B., Rodrigues W.F., Mendes M.T., de Oliveira C.J.F., Javier Chica E.L. (2015). Inflammatory responses and intestinal injury development during acute Trypanosoma cruzi infection are associated with the parasite load. Parasites Vectors.

[15] de Pinho R.T., da Silva W.S., de Castro Cortes L.M., da Silva Vasconcelos Sousa P., de Araujo Soares R.O., Alve C.R. (2014). Production of MMP-9 and inflammatory cytokines by *Trypanosoma cruzi*-infected macrophages. Exp. Parasitol..

[16] Abrankó L., García-Reyes J.F., Molina-Díaz A. (2011). In-source fragmentation and accurate mass analysis of multiclass flavonoid conjugates by electrospray ionization time-of-flight mass spectrometry. J. Mass Spectrom..

[17] Fujimoto K., Nakamura S., Matsumoto T., Ohta T., Yoshikawa M., Ogawa K., Kashiwazaki E., Matsuda H. (2014). Structures of acylated sucroses from the flower buds of *Prunus Mume*. J. Nat. Med..

[18] Nakamura S., Fujimoto K., Matsumoto T., Ohta T., Ogawa K., Tamura H., Matsuda H., Yoshikawa M. (2013). Structures of acylated sucroses and an acylated flavonol glycoside and inhibitory effects of constituents on aldose reductase from the flower buds of *Prunus mume*. J. Nat. Med..

[19] Shimazaki N., Mimaki Y., Sashida Y. (1991). Prunasin and acetylated phenylpropanoic acid sucrose esters, bitter principles from the fruits of *Prunus jamasakura* and *P. Maximowiczii*. Phytochemistry.

[20] Bonta R.K. (2017). Application of HPLC and ESI-MS techniques in the analysis of phenolic acids and flavonoids from green leafy vegetables (GLVs). J. Pharm. Anal..

[21] Kayano S., Kikuzaki H., Hashimoto S., Kasamatsu K., Ikami T., Nakatani N. (2014). Glucosyl terpenates from the dried fruits of *Prunus domestica* L.. Phytochem. Lett..

[22] Sanz M., de Simón B.F., Cadahía E., Esteruelas E., Muñoz A.M., Hernández T., Estrella I., Pinto E. (2012). LC-DAD/ESI-MS/MS study of phenolic compounds in ash (*Fraxinus excelsior* L. and *F. americana* L.) heartwood. Effect of toasting intensity at cooperage. J. Mass Spectrom..

[23] Zhao W., Huang X.-X., Yu L.-H., Liu Q.-B., Li L.-Z., Sun Q., Song S.-J. (2014). Tomensides A–D, new antiproliferative phenylpropanoid sucrose esters from *Prunus tomentosa* leaves. Bioorg. Med. Chem. Lett..

[24] Hölscher D., Schneider B. (1996). A resveratrol dimer from *Anigozanthos preissii* and *Musa Cavendish*. Phytochemistry.

[25] Ono M., Uenosono Y., Umaoka H., Shiono Y., Ikeda T., Okawa M., Kinjo J., Yoshimitsu H., Nohara T. (2009). Five New Steroidal Glycosides from the Stems of *Solanum sodomaeum*. Chem Pharm Bull..

[26] Guccione C., Bergonzi M.C., Piazzini V., Bilia A.R. (2016). A Simple and Rapid HPLC-PDA MS Method for the Profiling of *Citrus* Peels and Traditional Italian Liquors*. Planta Med..

[27] Zhang Y., Chen Y., Fan C., Ye W., Luo J. (2010). Two new iridoid glucosides from *Hedyotis diffusa*. Fitoterapia.

[28] Hussein S.R., Latif R.R.A., Marzouk M.M., Elkhateeb A., Mohammed R.S., Soliman A.A.F., Abdel-Hameed E.-S.S. (2018). Spectrometric analysis, phenolics isolation and cytotoxic activity of *Stipagrostis* plumosa (Family Poaceae). Chem Pap..

[29] Shan M., Yu S., Yan H., Guo S., Xiao W., Wang Z., Zhang L., Ding A., Wu Q., Li S.F.Y. (2017). A Review on the Phytochemistry, Pharmacology, Pharmacokinetics and Toxicology of Geniposide, a Natural Product. Molecules.

[30] Chang C.L., Zhang L.J., Chen R.Y., Kuo L.M., Huang J.P., Huang H.C., Lee K.H., Wu Y.C., Kuo Y.H. (2010). Antioxidant and anti-inflammatory phenylpropanoid derivatives from *Calamus quiquesetinervius*. J. Nat. Prod..

[31] Dawé A., Mbiantcha M., Yakai F., Jabeen A., Ali M.S., Lateef M., Ngadjui B.T. (2018). Flavonoids and triterpenes from *Combretum fragrans* with anti-inflammatory, antioxidant and antidiabetic potential. Z Nat. C..

[32] Rao Y.K., Fang S.H., Tzeng Y.M. (2008). Anti-inflammatory activities of flavonoids and a triterpene caffeate isolated from *Bauhinia variegata*. Phytother Res..

[33] Deng Y.H., Alex D., Huang H.Q., Wang N., Yu N., Wang Y.T., Leung G.P., Lee S.M. (2011). Inhibition of TNF-α-mediated endothelial cell-monocyte cell adhesion and adhesion molecules expression by the resveratrol derivative, trans-3,5,4’-trimethoxystilbene. Phytother Res..

[34] An S.J., Pae H.O., Oh G.S., Choi B.M., Jeong S., Jang S.I., Oh H., Kwon T.O., Song C.E., Chung H.T. (2002). Inhibition of TNF-alpha, IL-1beta, and IL-6 productions and NF-kappa B activation in lipopolysaccharide-activated RAW 264.7 macrophages by catalposide, an iridoid glycoside isolated from *Catalpa ovata* G. *Don (Bignoniaceae)*. Int. Immunopharmacol..

[35] Kim M.K., Yun K.J., Lim D.H., Kim J., Jang Y.P. (2016). Anti-Inflammatory Properties of Flavone di-C-Glycosides as Active Principles of *Camellia Mistletoe*, *Korthalsella japonica*. Biomol..

[36] Gutierrez F.R.S., Mineo T.W.P., Pavanelli W.R., Guedes P.M.M., Silva J.S. (2009). The effects of nitric oxide on the immune system during *Trypanosoma cruzi* infection. Mem. Inst. Oswaldo Cruzrio De Jan..

[37] Silva A.M., Machado I.D., Santin J.R., de Melo I.L., Pedrosa G.V., Genovese M.I., Farsky S.H., Mancini-Filho J. (2015). Aqueous extract of *Rosmarinus officinalis* L. inhibits neutrophil influx and cytokine secretion. Phytother Res..

[38] Flecknell P. (2002). Replacement, reduction and refinement. ALTEX.

[39] Schwende H., Fitzke E., Ambs P., Dieter P. (1996). Differences in the state of differentiation of THP-1 cells induced by phorbol ester and 1,25-dihydroxyvitamin D3. J. Leukoc Biol..

[40] Buckner F.S., Verlinde C.L., La Flamme A.C., Van Voorhis W.C. (1996). Efficient technique for screening drugs for activity against *Trypanosoma cruzi* using parasites expressing β-galactoside. Antimicrob. Agents Chemother.

[41] Sieuwerts A.M., Klijn J.G.M., Peters H.A., Foekens J.A. (1995). The MTT tetrazolium salt assay scrutinized: How to use this assay reliably to measure metabolic activity of cell cultures in vitro for the assessment of growth characteristics, IC_50_-values and cell survival. Clin. Chem. Lab. Med..

[42] Van de Loosdrecht A., Nennie E., Ossenkoppele G., Beelen R., Langenhuijsen M. (1991). Cell mediated cytotoxicity against U 937 cells by human monocytes and macrophages in a modified colorimetric MTT assay. A Methodol. Study. J. Immunol. Methods.

