# Supplementary material for: NOx-, IL-1β-, TNF-α-, and IL-6-Inhibiting Effects and Trypanocidal Activity of Banana (Musa acuminata) Bracts and Flowers: UPLC-HRESI-MS Detection of Phenylpropanoid Sucrose Esters
Source: Molecules. 2019 Dec 13;24(24):4564. doi: 10.3390/molecules24244564 (PMC6943641; doi:10.3390/molecules24244564)
Supplement: Supplementary file 1 [file molecules-24-04564-s001.pdf]

Supporting information

NOx, IL-1β, TNF-α, IL-6 inhibition effects and trypanocidal activity of banana (*Musa acuminata*) bracts and flowers: UPLC-HRESI-MS detection of phenylpropanoid sucrose esters.

Louis P. Sandjo [1,*] Marcus V. P. dos Santos Nascimento [2], Milene de H. Moraes[3], Luiza M. Rodrigues [3], Eduardo M. Dalmarco[2], Maique W. Biavatti[4] and Mario Steindel[3,]**

[1] Department of Chemistry, Federal University of Santa Catarina, 88040-900 Florianópolis, SC, Brazil, p.l.sandjo@ufsc.br (L.P.S.)

[2] Department of Clinical Analysis, Centre of Health Sciences, Federal University of Santa Catarina, Florianópolis, Brazil; mmmarcusster@gmail.com (M.V.P.S.N.); eduardo.dalmarco@ufsc.br (E.M.D.)

[3] Laboratory of Protozoology, Department of Microbiology, Immunology and Parasitology Centre of biological sciences, Federal University of Santa Catarina, Florianópolis, Brazil; milenehoehr@gmail.com (M.H.M.); luizamanaut@gmail.com (L.M.R); mario.steindel@ufsc.br (M.S.)

[4] Department of Pharmaceutical Sciences, Centre of Health Sciences, Federal University of Santa Catarina, Florianópolis, Brazil. maique.biavatti@ufsc.br (M.W.B)

* Correspondence: E-mail: p.l.sandjo@ufsc.br; Tel: +55483721-3624 (L.P.S); mario.steindel@ufsc.br; Tel: +55483721-2958 (M.S.)

Table S1. Chemical constituents of the flower fractions.

FDCM						
**tR**	**BPI (m/z)**	**Mass calculated (m/z)**	**Molecular formula**	**Fragment**	Proposed structure	**Ref.**
6.66	613.1731 [M-H]$^-$	613.1769	$C_{27}H_{34}O_{16}$	571.1544	Tomenside A, mumeose G, mumeose S or mumeose H	[1-3]
6.99	613.1731 [M-H]$^-$	613.1769	$C_{27}H_{34}O_{16}$	571.1544	Tomenside A, mumeose G, mumeose S or mumeose H	[1-3]
7.32	655.1882 [M-H]$^-$	655.1874	$C_{29}H_{36}O_{17}$	613.1680, 595.1530, 571.1544, 553.1459, 467.1384, 425.1317, 349.0909, 187.0343, 163.0352, 145.0278	mumeose L or mumeose U	[2, 4]
7.58	655.1882 [M-H]$^-$	655.1874	$C_{29}H_{36}O_{17}$	613.1680, 595.1580, 571.1544, 553.1363, 535.1274, 467.1340, 425.1233, 391.0948, 383.1170, 349.0832, 307.0778, 289.0623, 217.0513, 205.0422, 187.0343, 163.0352, 145.0278, 119.0473	4,6,2',6'-O-tetraacetyl-3-O-cis-p-coumaroylsucrose or 4,6,2',6'-O-tetraacetyl-3-O-trans-p-coumaroylsucrose	[5]
7.94	697.1997 [M-H]$^-$	697.1980	$C_{31}H_{38}O_{18}$	655.1882, 637.1663, 467.1251, 349.0870, 163.0378, 145.0278	mumeose V or mumeose D	[2]
8.05	697.1997 [M-H]$^-$	697.1980	$C_{31}H_{38}O_{18}$	655.1852, 637.1766, 613.1680, 595.1580, 467.1340, 425.1486, 391.1028, 349.0870, 205.0480, 187.0343, 163.0352, 145.0278	Prunose I or mumeose N, or mumeose M or mumeose O	[4]
8.16	697.1997 [M-H]$^-$	697.1980	$C_{31}H_{38}O_{18}$	655.1882, 637.1663, 613.1680, 595.1580, 467.1340, 425.1567, 391.1028, 349.1176, 205.0480, 187.0371, 163.0352, 145.0254	Prunose I or mumeose N, or mumeose M or mumeose O	[4]
8.27	697.1997 [M-H]$^-$	697.1980	$C_{31}H_{38}O_{18}$	655.1882, 637.1714, 613.1730, 595.1580, 467.1517, 391.0988, 349.0870, 205.0480,	Prunose I or mumeose N, or mumeose M or mumeose O	[4]

				187.0371, 163.0352, 145.0278		
8.75	221.1191 [M-H]-	221.1178	$C_{13}H_{18}O_3$	-	Alkylated phenol derivative	
10.80	277.1817 [M-H]-	277.1804	$C_{17}H_{26}O_3$	-	NI	
11.02	265.1468 [M-H]-	265.1440	$C_{15}H_{22}O_4$	-	NI	
14.22	279.2312 [M-H]-	279.2324	$C_{18}H_{32}O_2$	-	Linoleic acid	[6]
14.69	255.2306 [M-H]-	255.2324	$C_{16}H_{32}O_2$	-	1-hexadecylcarboxylic acid	[7]
14.80	281.2470 [M-H]-	281.2481	$C_{18}H_{34}O_2$	-	Related to petroselinic acid	[8]
15.68	283.2680 [M-H]-	283.2637	$C_{18}H_{36}O_2$	-	n-octadecanoic acid	[9]
FNBU						
0.49	191.0551 [M-H]-	191.0561	$C_7H_{12}O_6$	-	Quinic acid	[10]
2.18	487.1465 [M-H]-	487.1457	$C_{21}H_{28}O_{13}$	307.0706, 163.0431, 145.0352	3-O-*p*-coumaroylsucrose	[11]
2.37	487.1465 [M-H]-	487.1457	$C_{21}H_{28}O_{13}$	341.0868, 179.0580	Cistanoside F	[12]
3.43	529.1561 [M-H]-	529.1563	$C_{23}H_{30}O_{14}$	487.1449, 469.1057, 341.0905	Acetyl cistanoside F derivative	-
3.69	529.1561 [M-H]-	529.1563	$C_{23}H_{30}O_{14}$	487.1330, 307.0742, 163.0405, 145.0352	mumeose A derivative	[13]
4.35	529.1561 [M-H]-	529.1563	$C_{23}H_{30}O_{14}$	487.1330, 163.0352, 145.0254	mumeose A derivative	[13]
4.60	571.1593 [M-H]-	571.1663	$C_{25}H_{32}O_{15}$	529.1467, 511.1470, 307.0814, 163.0405, 145.0352	mumeose B, P, or R	[2, 13]
5.15	571.1642 [M-H]-	571.1663	$C_{25}H_{32}O_{15}$	529.1467, 511.1470, 307.0814, 163.0405, 145.0352	mumeose B, P, or R	[2, 13]
5.41	571.1671 [M-H]-	571.1663	$C_{25}H_{32}O_{15}$	529.1514, 511.1470, 307.0778, 163.0352, 145.0354	mumeose B, P, or R	[2, 13]
5.70	571.1642 [M-H]-	571.1663	$C_{25}H_{32}O_{15}$	529.1467, 511.1470, 307.0778, 163.0405, 145.0354	mumeose B, P, or R	[2, 13]
6.07	613.1731 [M-H]-	613.1769	$C_{27}H_{34}O_{16}$	571.1592, 553.1507, 529.1467, 511.1423, 349.0909, 307.0814, 163.0378, 145.0278	Tomenside B derivative	[3]
6.18	613.1731	613.1769	$C_{27}H_{34}O_{16}$	571.1592, 553.1507,	Tomenside B derivative	[3]

	[M-H]-			529.1467, 511.1423, 349.0909, 307.0814, 163.0378, 145.0278		
6.40	613.1731 [M-H]-	613.1769	$C_{27}H_{34}O_{16}$	571.1592, 553.1507, 529.1467, 511.1423, 349.0909, 307.0814, 163.0378, 145.0278	Tomenside B derivative	[3]
6.66	613.1731 [M-H]-	613.1769	$C_{27}H_{34}O_{16}$	571.1592, 553.1507, 529.1467, 511.1423, 349.0909, 307.0814, 163.0378, 145.0278	Tomenside B derivative	[3]
6.99	613.1731 [M-H]-	613.1769	$C_{27}H_{34}O_{16}$	571.1592, 553.1507, 529.1467, 511.1423, 349.0909, 307.0814, 163.0378, 145.0278	Tomenside B derivative	[3]
7.32	655.1882 [M-H]-	655.1874	$C_{29}H_{36}O_{17}$	613.1680, 595.1530, 571.1544, 553.1459, 467.1384, 425.1317, 349.0909, 205.0422, 187.0343, 163.0352, 145.0278	mumeose L or mumeose U	[2, 4]
7.58	655.1882 [M-H]-	655.1874	$C_{29}H_{36}O_{17}$	613.1680, 595.1580, 571.1544, 553.1363, 535.1274, 467.1340, 425.1233, 391.0948, 383.1170, 349.0832, 307.0778, 289.0623, 217.0513, 205.0422, 187.0343, 163.0352, 145.0278, 119.0473	4,6,2',6'-O-tetraacetyl-3-O-cis-p-coumaroylsucrose or 4,6,2',6'-O-tetraacetyl-3-O-trans-p-coumaroylsucrose	[5]
8.75	221.1191 [M-H]-	221.1178	$C_{13}H_{18}O_3$	-	Alkylated phenol derivative	-
11.80	1197.2529 [2M+HCO2]-	1197.2512	$C_{32}H_{24}O_{12}$	-	Procyanidin derivatives	-
12.60	637.1405 [M+HCO2]-	637.1405	$C_{27}H_{28}O_{15}$	-	Glycosylated flavonoid	-
13.45	455.3515 [M-H]-	455.3525	$C_{30}H_{48}O_3$	-	Ursolic acid, betulenic acid or oleanolic acid	-
14.07	609.4099 [M-H]-	609.4049	$C_{39}H_{56}O_5$	-	Betuline-3-caffeate or erythrodiol-3-caffeate	-

NI: not identified

Table S2. Chemical constituents of the bract fractions.

tR	BPI (m/z)	Mass calculated (m/z)	Molecular formula	Fragment	Proposed structure	Ref.
**BDCM**						
7.36	177.0550 [M-H]-	177.0552	$C_{10}H_{10}O_3$	-	Coumaric acid methyl ester or 4-methoxycinnamic acid	[14]
10.69	721.3635 [M+HCO2]- 675.3600 [M-H]-	721.3647 675.3597	$C_{33}H_{56}O_{14}$	593.3170	Physakengose B	[15]
11.54	723.3801 [M+HCO2]- 677.3730 [M-H]-	723.3803 677.3690	$C_{33}H_{58}O_{14}$	595.2879, 397.1343	3'-O-isobutyryl-3-O-isovaleryl-2-O-lauroylsucrose or 2,3,4-tri(5-methylhexanoyl)-α-D-glucopyranosyl-β-D-fructofuranoside	[16]
11.76	559.3087 [M+HCO2]-	559.3060	$C_{34}H_{44}O_4$	-	Stilbene derivative	-
12.57	445.2352 [M-H]-	445.2379	$C_{29}H_{34}O_4$	-	2-Arylbenzofuran derivative	-
13.78	447.2509 [M-H]-	447.2535	$C_{29}H_{36}O_4$	-	Stilbene derivative	-
14.22	279.2312 [M-H]-	279.2324	$C_{18}H_{32}O_2$	-	Linoleic acid derivative	[6]
14.66	255.2339 [M-H]-	255.2324	$C_{16}H_{32}O_2$	-	Fatty acid	-
BNBU						
2.29	293.1223 [M-H]-	293.1236	$C_{12}H_{22}O_8$	-	γ-methyl-δ-hydroxy-pentanoic acid β-D-glucopyranoside	-
3.76	431.1871 [M-H]-	431.1858	$C_{27}H_{28}O_5$	-	NI	-
4.05	377.1757 [M-H]-	377.1753	$C_{24}H_{26}O_4$	-	Benzofuran derivative	-
5.01	609.1482 [M-H]-	609.1456	$C_{27}H_{30}O_{16}$	581.1510, 461.1089	Lucenin-2	[17]
7.87	431.1531 [M-H]-	431.1553	$C_{19}H_{28}O_{11}$	349.0985, 331.0892, 113.0320	Diffusoside A or B	[18]
8.05	433.1710 [M-H]-	433.1710	$C_{19}H_{30}O_{11}$	351.1140, 333.1045, 267.0643,	7-O-Ethylmorroniside	[19]

				249.0587, 113.0298, 101.0698		
8.31	435.1851 $[M-H]^-$	435.1866	$C_{19}H_{32}O_{11}$	375.1381, 351.1102, 333.1045, 249.0490, 223.0397, 175.0307, 113.0298, 101.0698	Iridoid derivative	-
8.75	221.1191 $[M-H]^-$	221.1178	$C_{13}H_{18}O_3$	-	Alkylated phenol derivative	-
13.23	447.2509 $[M-H]^-$	447.2535	$C_{29}H_{36}O_4$	-	Stilbene derivative	-

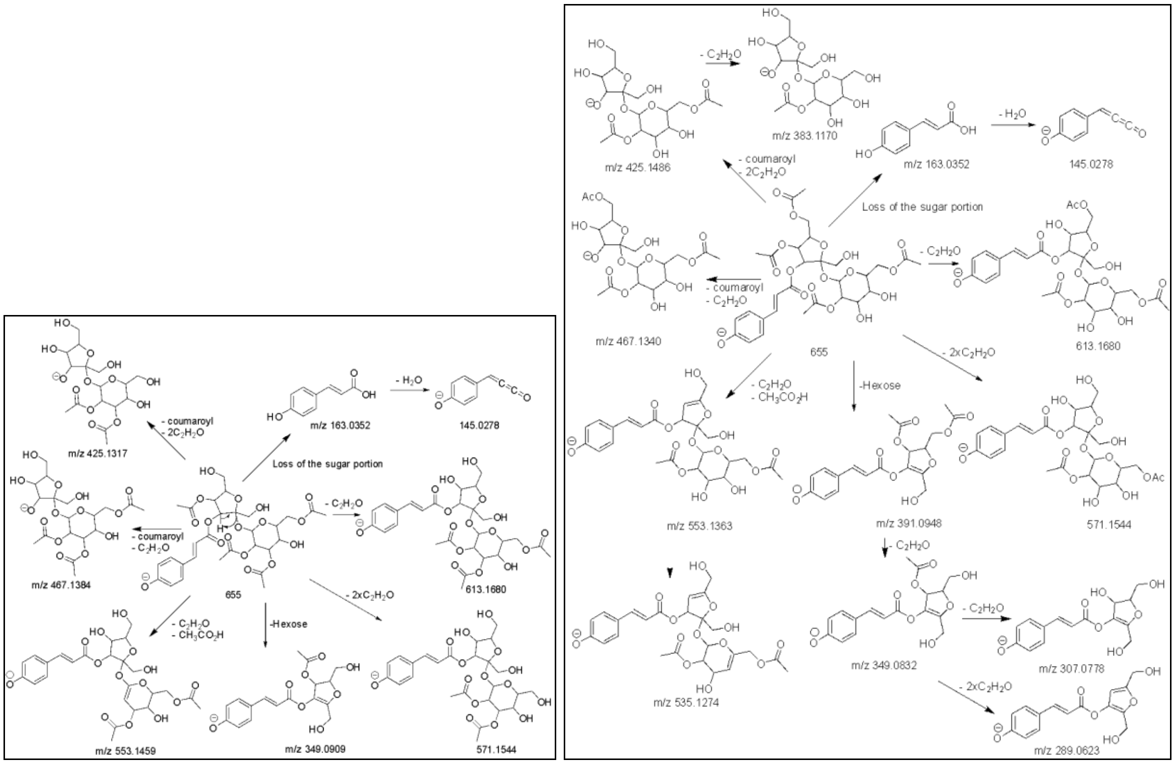

Figure S1. Fragmentation pattern of the metabolites m/z 655.1874 with retention times 7.32 min (left) and 7.58 min (right).

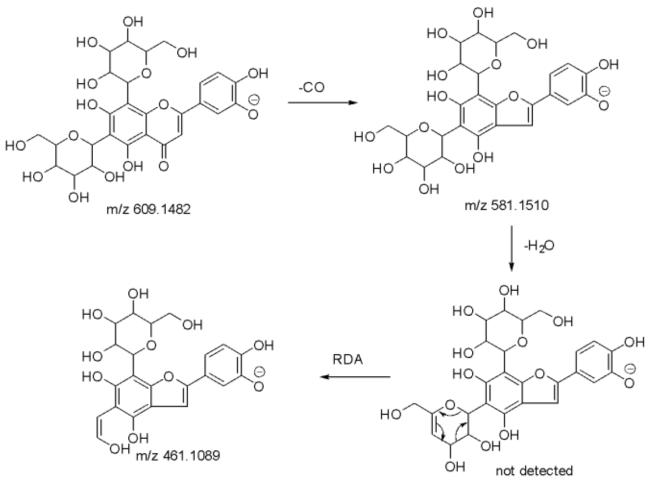

Figure S2. Fragmentation pattern of the metabolite m/z 609.1482.

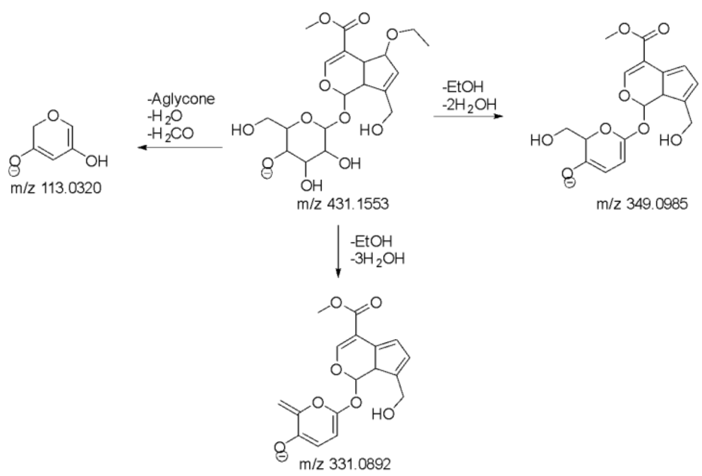

Figure S3. Fragmentation pattern of m/z 431.1553.

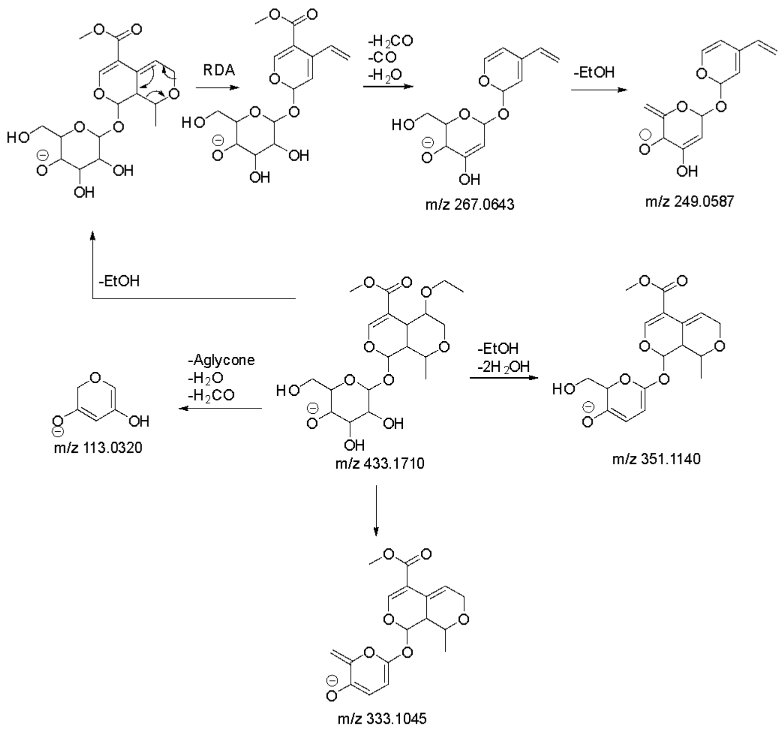

Figure S4. Fragmentation pattern of m/z 433.1710.

References

1. Fujimoto, K.; Nakamura, S.; Matsumoto, T.; Ohta, T.; Ogawa, K.; Tamura, H.; Matsuda, H.; Yoshikawa, M. Medicinal flowers. XXXVIII. structures of acylated sucroses and inhibitory effects of constituents on aldose reducatase from the flower buds of *Prunus mume*. Chem Pharm Bull (Tokyo). **2013**, *61*, 445-451. doi: 10.1248/cpb.c12-01068.

2. Fujimoto, K.; Nakamura, S.; Matsumoto, T.; Ohta, T.; Yoshikawa, M.; Ogawa, K.; Kashiwazaki, E.; Matsuda, H. Structures of acylated sucroses from the flower buds of *Prunus mume.* J Nat Med, **2014**, *68*, 481-487, doi: 10.1007/s11418-014-0818-z.

3. Zhao, W.; Huang, X.-X.; Yu, L.-H.; Liu, Q.-B.; Li, L.-Z.; Sun, Q.; Song, S.-J. Tomensides A–D, new antiproliferative phenylpropanoid sucrose esters from *Prunus tomentosa* leaves. Bioorg Med Chem Lett, **2014**, *24*, 2459–2462, doi: 10.1016/j.bmcl.2014.04.018

4. Nakamura, S.; Fujimoto, K.; Matsumoto, T.; Ohta, T.; Ogawa, K.; Tamura, H.; Matsuda, H. Yoshikawa, M. Structures of acylated sucroses and an acylated flavonol glycoside and inhibitory effects of constituents on aldose reductase from the flower buds of *Prunus mume*. J Nat Med. **2013,** *67*, 799-806. doi: 10.1007/s11418-013-0750-7

5. Shimazaki, N.; Mimaki, Y.; Sashida, Y. Prunasin and acetylated phenylpropanoic acid sucrose esters, bitter principles from the fruits of *Prunus jamasakura* and *P. Maximowiczii.* Phytochemistry **1991**, *30*, 1475-1480, doi: 10.1016/0031-9422(91)84190-4

6. Gao, S.Y.; Gong, Y.F.; Sun, Q.J.; Bai, J.; Wang, L.; Fan, Z.Q.; Sun, Y.; Su, Y.J.; Gang, J.; Ji, Y.B. Screening Antitumor Bioactive Fraction from *Sauromatum giganteum* (Engl.) Cusimano & Hett and Sensitive Cell Lines with the Serum Pharmacology Method and Identification by UPLC-TOF-MS. Molecules **2015**, *20*, 4290-4306; doi:10.3390/molecules20034290.

7. Rajput, A.P.; Patil B.S. Phytochemical Analysis of Saponifiable Matter of Petroleum Ether Extract of Leaves of *Butea monosperma.* Asian J Chem, **2013**, *25*, 6016-6020, doi: 10.14233/ajchem.2013.14236.

8. Goyal, S.; Banerjee, S.; Mazumdar, S. Oxygenation of monoenoic fatty acids by CYP175A1, an orphan cytochrome P450 from *Thermus thermophilus* HB27. Biochemistry. **2012**, *51*, 7880-7890. doi: 10.1021/bi300514j

9. Rahman, A.F.; Angawi, R.F.; Kadi, A.A. Spatial localisation of curcumin and rapid screening of the chemical compositions of turmeric rhizomes (*Curcuma longa* Linn.) using Direct Analysis in Real Time-Mass Spectrometry (DART-MS). Food Chem. 2015, 173, 489-494. doi: 10.1016/j.foodchem.2014.10.049.

10. Dawidowicz, A.L.; Typek, R. Transformation of 5-O-caffeoylquinic acid in blueberries during high-temperature processing. J Agric Food Chem. **2014**, *62*, 10889-10895. doi: 10.1021/jf503993q.

11. Kayano, S. Kikuzaki, H.; Hashimoto, S.; Kasamatsu, K.; Ikami, T.; Nakatani, N. Glucosyl terpenates from the dried fruits of *Prunus domestica* L. Phytochem Lett, **2014**, *8*, 132–136, doi: 10.1016/j.phytol.2014.03.006

12. Sanz, M.; de Simón, B.F.; Cadahía, E.; Esteruelas, E.; Muñoz, A.M.; Hernández, T.; Estrella, I.; Pinto, E. LC-DAD/ESI-MS/MS study of phenolic compounds in ash (*Fraxinus excelsior* L. and *F. americana* L.) heartwood. Effect of toasting intensity at cooperage. J Mass Spectrom, **2012**, *47*, 905-918, doi: 10.1002/jms.3040.

13. Nakamura, S.; Fujimoto, K.; Matsumoto, T.; Nakashima, S.; Ohta, T.; Ogawa, K.; Matsuda, H.; Yoshikawa, M. Acylated sucroses and acylated quinic acids analogs from the flower buds of *Prunus mume* and their inhibitory effect on melanogenesis. Phytochemistry. **2013**, *92*, 128-136. doi: 10.1016/j.phytochem.2013.04.012.

14. Cruz, M.; Antunes, P.; Paulo, L.; Ferreira,A.M.; Cunha, A.; Almeida-Aguiara, C.; Oliveira, R. Antioxidant and dual dose-dependent antigenotoxic and genotoxic properties of an ethanol extract of propolis, RSC Adv., **2016**, *6*, 49806-49816, doi: 10.1039/C6RA04856K.

15. Zhang, C.Y.; Luo, J.G.; Liu, R.H.; Lin, R.; Yang, M.H.; Kong, L.Y. ^{1}H NMR spectroscopy-guided isolation of new sucrose esters from *Physalis alkekengi* var. franchetii and their antibacterial activity. Fitoterapia **2016**, *114*, 138-143. doi: 10.1016/j.fitote.2016.09.007.

16. Pérez-Castorena, A.L.; Luna, M.; Martínez, M.; Maldonado E. New sucrose esters from the fruits of *Physalis solanaceus*. Carbohydr Res. **2012**, *352*, 211-214. doi: 10.1016/j.carres.2012.02.003.

17. Simirgiotis, M.J.; Schmeda-Hirschmann, G.; Bórquez, J.; Kennelly, E.J. The *Passiflora tripartita* (Banana Passion) Fruit: A Source of Bioactive Flavonoid C-Glycosides Isolated by HSCCC and Characterized by HPLC–DAD–ESI/MS/MS. Molecules. **2013**, *18*, 1672-192. doi: 10.3390/molecules18021672.

18. Zhang, Y.; Chen, Y.; Fan, C.; Ye, W.; Luo, J. Two new iridoid glucosides from *Hedyotis diffusa*. Fitoterapia **2010**, *81*, 515–517, doi: 10.1016/j.fitote.2010.01.010.

19. Wang, L.; Chen, H.; Jiang, Y.; Liu, Z.; Wang, Q.; Zheng, X. Simultaneous Determination of 11 High-Polarity Components from *Fructus Corni*: A Quantitative LC–MS/MS Method for Improved Quality Control. J Chromatogr Sci. 2018, 56, 56-64. doi: 10.1093/chromsci/bmx083
